# *In silico* identification of vaccine targets for 2019-nCoV

**DOI:** 10.12688/f1000research.22507.2

**Published:** 2020-04-14

**Authors:** Chloe H. Lee, Hashem Koohy

**Affiliations:** 1 MRC Human Immunology Unit, Medical Research Council (MRC) Human Immunology Unit, MRC Weatherall Institute of Molecular Medicine (WIMM), John Radcliffe Hospital, University of Oxford, Oxford, UK, Oxford, UK

**Keywords:** Coronavirus, adaptive immunity, immunogenicity, T cell cross-reactivity, vaccine development

## Abstract

**Background:** The newly identified coronavirus known as 2019-nCoV has posed a serious global health threat. According to the latest report (18-February-2020), it has infected more than 72,000 people globally and led to deaths of more than 1,016 people in China.

**Methods:** The 2019 novel coronavirus proteome was aligned to a curated database of viral immunogenic peptides. The immunogenicity of detected peptides and their binding potential to HLA alleles was predicted by immunogenicity predictive models and NetMHCpan 4.0.

**Results:** We report *in silico* identification of a comprehensive list of immunogenic peptides that can be used as potential targets for 2019 novel coronavirus (2019-nCoV) vaccine development. First, we found 28 nCoV peptides identical to Severe acute respiratory syndrome-related coronavirus (SARS CoV) that have previously been characterized immunogenic by T cell assays. Second, we identified 48 nCoV peptides having a high degree of similarity with immunogenic peptides deposited in The Immune Epitope Database (IEDB). Lastly, we conducted a *de novo* search of 2019-nCoV 9-mer peptides that i) bind to common HLA alleles in Chinese and European population and ii) have T Cell Receptor (TCR) recognition potential by positional weight matrices and a recently developed immunogenicity algorithm, iPred, and identified in total 63 peptides with a high immunogenicity potential.

**Conclusions:** Given the limited time and resources to develop vaccine and treatments for 2019-nCoV, our work provides a shortlist of candidates for experimental validation and thus can accelerate development pipeline.

## Introduction

The emergence and rapid spread of the recent novel coronavirus known as 2019-nCoV has posed a serious global health threat ^1^ and has already caused a huge financial burden ^2^. It has further challenged the scientific and industrial community for quick control practices, and equally importantly to develop effective vaccines to prevent its recurrence. In facing a rapid epidemical outbreak to a novel and unknown pathogen, a key bottleneck for a proper and deep investigation, which is fundamental for vaccine development, is the limited -- to almost no -- access of the scientific community to samples from infected subjects. As such, *in silico* predictions of targets for vaccines are of high importance and can serve as a guidance to medical and experimental experts for the best and timely use of the limited resources.

In this regard, we report our recent effort to computationally identify immunogenic and/or cross-reactive peptides from 2019-nCoV. We provide a detailed screen of candidate peptides based on comparison with immunogenic peptides deposited in the Immune Epitope Database and Analysis Resource (IEDB) database including those derived from Severe acute respiratory syndrome-related coronavirus (SARS CoV) along with *de novo* prediction from 2019-nCoV 9-mer peptides. Here, we found i) 28 SARS-derived peptides having exact matches in 2019-nCoV proteome previously characterized to be immunogenic by *in vitro* T cell assays, ii) 22 nCoV peptides having a high sequence similarity with immunogenic peptides but with a greater predicted immunogenicity score, and iii) 44 + 19 nCoV peptides predicted to be immunogenic by the iPred algorithm and 1G4 TCR positional weight matrices respectively.

## Results

### Identification of 28 exact matches to SARS CoV immunogenic peptides by screening all epitopes deposited in IEDB

We collected all peptides in IEDB ( 3, as of 13-02-2020) reported positive in T cell assays and have human as the host organism. We then conducted a local sequence alignment of 10 2019-nCoV open reading frames (ORFs) against 35,225 IEDB peptides, and found 28 exact matches. Surprisingly, all identical hits (against peptides having sequence length greater than 3) were from SARS-CoV ( Table 1, Data Table 1 ^4^). These peptides have been shown to bind various HLA alleles, although with higher tendency towards HLA-A:02:01, from both class I and class II, and can be target for CD8+ and CD4+ T cells respectively.

**Table 1.** 28 2019-nCoV peptides having exact matches with immunogenic SARS-CoV peptides.

IEDB.peptide	2019-nCoV.pattern	Antigen.Name	Allele.Name
TLACFVLAAV	TLACFVLAAV	Membrane glycoprotein	HLA-A *02:01
AFFGMSRIGMEVTPSGTW	AFFGMSRIGMEVTPSGTW	N protein	
ALNTPKDHI	ALNTPKDHI	Nucleoprotein	HLA-A *02:01
AQFAPSASAFFGMSR	AQFAPSASAFFGMSR	nucleocapsid protein	HLA class II
AQFAPSASAFFGMSRIGM	AQFAPSASAFFGMSRIGM	N protein	
GMSRIGMEV	GMSRIGMEV	Nucleoprotein	HLA-A *02:01
ILLNKHIDA	ILLNKHIDA	Nucleoprotein	HLA-A *02:01
IRQGTDYKHWPQIAQFA	IRQGTDYKHWPQIAQFA	N protein	
KHWPQIAQFAPSASAFF	KHWPQIAQFAPSASAFF	N protein	
LALLLLDRL	LALLLLDRL	Nucleoprotein	HLA-A *02:01
LLLDRLNQL	LLLDRLNQL	Nucleoprotein	HLA-A *02:01
LLNKHIDAYKTFPPTEPK	LLNKHIDAYKTFPPTEPK	N protein	
LQLPQGTTL	LQLPQGTTL	Nucleoprotein	HLA-A *02:01
RRPQGLPNNTASWFT	RRPQGLPNNTASWFT	nucleocapsid protein	HLA class I
YKTFPPTEPKKDKKKK	YKTFPPTEPKKDKKKK	N protein	
ILLNKHID	ILLNKHID	Nucleoprotein	HLA-A *02:01
MEVTPSGTWL	MEVTPSGTWL	nucleocapsid protein	HLA-B *40:01
ALNTLVKQL	ALNTLVKQL	S protein	HLA-A *02:01
FIAGLIAIV	FIAGLIAIV	Spike glycoprotein precursor	HLA-A2
LITGRLQSL	LITGRLQSL	Spike glycoprotein precursor	HLA-A2
NLNESLIDL	NLNESLIDL	S protein	HLA-A *02:01
QALNTLVKQLSSNFGAI	QALNTLVKQLSSNFGAI	S protein	HLA-DRB1 *04:01
RLNEVAKNL	RLNEVAKNL	Spike glycoprotein precursor	HLA-A *02:01
VLNDILSRL	VLNDILSRL	S protein	HLA-A *02:01
VVFLHVTYV	VVFLHVTYV	Spike glycoprotein precursor	HLA-A *02:01
GAALQIPFAMQMAYRF	GAALQIPFAMQMAYRF	S protein	HLA-DRA *01:01/DRB1 *07:01
MAYRFNGIGVTQNVLY	MAYRFNGIGVTQNVLY	S protein	HLA-DRB1 *04:01
QLIRAAEIRASANLAATK	QLIRAAEIRASANLAATK	S protein	HLA-DRB1 *04:01

*SARS-CoV: Severe acute respiratory syndrome coronavirus

### Identification of 22 2019-nCoV peptides with high degree of similarity to previously reported immunogenic viral peptides

**Figure 1.**
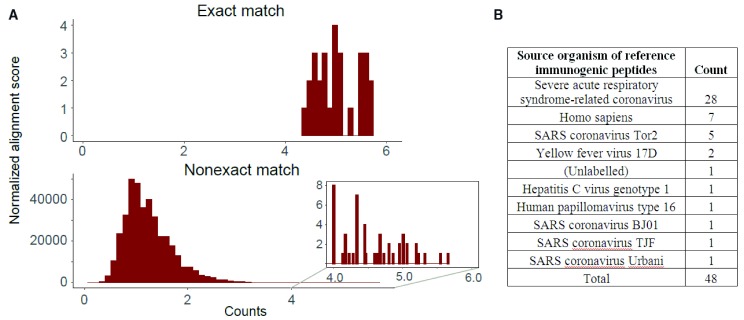
2019-nCoV peptides with high sequence similarity to immunogenic peptides in IEDB. **A.** Comparison of normalized sequence alignment score for peptides with exact and non-exact matches. **B.** Number of target peptides grouped by their source organism.

In addition to 28 identical hits against SARS CoV, we observed a long tail in distribution of normalized alignment scores between 10 2019-nCoV ORFs and 35,225 IEDB peptides ( Figure 1A, Methods). We therefore set out to further investigate potential vaccine targets among highly similar sequences.

The peptides having an exact sequence alignment with epitopes in IEDB had normalized alignment scores ranging from 4 to 6. Taking the normalized alignment score of exact matches as a reference, we extracted 2019-nCoV peptides having score greater or equal to 4. As illustrated in Figure 1A, we observed 45 and 11 peptides having normalized alignment score ≥ 4 and ≥ 5 respectively ( Figure 1A inset). The target peptides were originated from 10 different sources ( Figure 1B) where a total 36 peptides were derived from strains associated to SARS CoV. Of interest, we also observed 7 hits having high sequence similarity to targets from *Homo sapiens*.

In order to investigate the extent to which the difference between the source (2019-nCoV) and target (IEDB) peptides influences the immunogenicity of the source peptides we used a recently published immunogenicity model ^5^ to predict and compare the immunogenicity between the source and target peptides (Data Table 2 ^4^).

**Figure 2.**
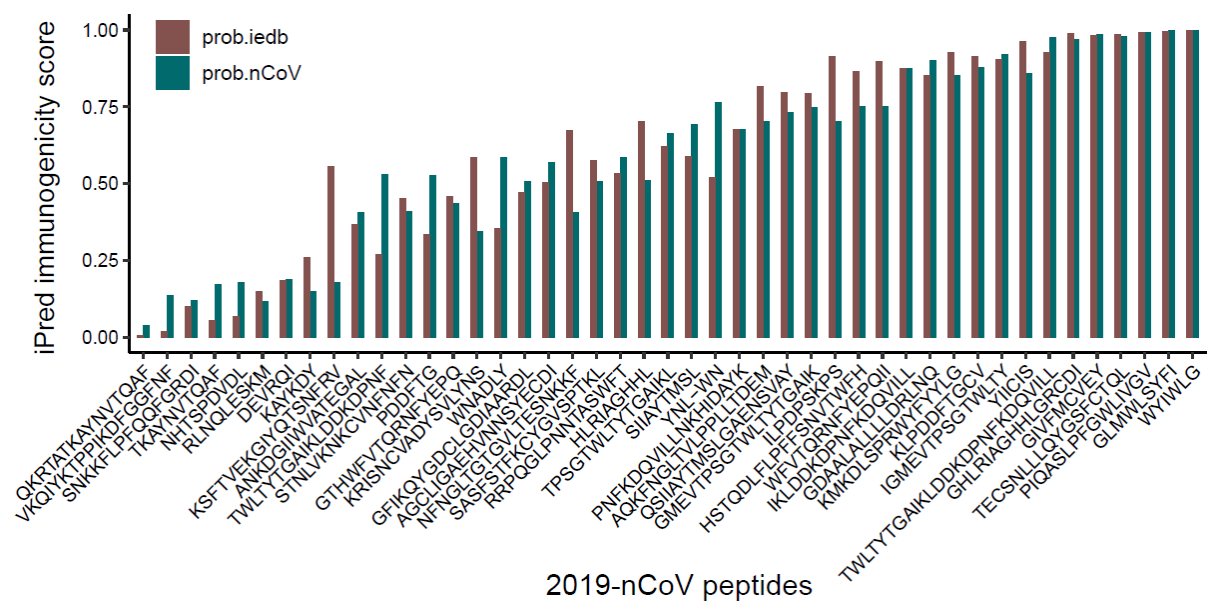
Predicted immunogenicity for IEDB immunogenic vs. 2019-nCoV peptides. 2019-nCoV peptides having a high sequence similarity to immunogenic peptides and their targets were analysed for their immunogenicity potential by iPred algorithm.

We could see a similar (close to identical) immunogenicity scores for a number of IEDB and 2019-nCov peptides especially for those with high immunogenicity scores ( Figure 2). While all 48 can be potential targets, of particular interest were those having higher immunogenicity score than IEDB peptides. Here, we list 22 out of 48 2019-nCoV peptides that scored higher compared to their targets that have been characterized to be immunogenic ( Table 2). In this list 15 (68%) 2019-nCov peptides have a score higher than 0.5 whereas only 11(50%) of IEDB get a score immunogenicity score greater than 0.5.

**Table 2.** List of 22 2019-nCoV peptides having a higher predicted immunogenicity score than their target peptides.

IEDB.peptide	2019-nCoV.pattern	IEDB.prob	nCol.prob
WYMWLGARY	WYIWLG	0.999249	0.999441
GLMWLSYFV	GLMWLSYFI	0.995073	0.998216
GLVFLCLQY	GIVFMCVEY	0.98123	0.984127
TWLTYHGAIKLDDKDPQFKDNVILL	TWLTYTGAIKLDDKDPNFKDQVILL	0.925862	0.975242
IGMEVTPSGTWLTYH	IGMEVTPSGTWLTY	0.903518	0.919184
GETALALLLLDRLNQ	GDAALALLLLDRLNQ	0.853114	0.900655
TPSGTWLTYHGAIKL	TPSGTWLTYTGAIKL	0.620894	0.662417
SIVAYTMSL	SIIAYTMSL	0.589694	0.693763
RRPQGLPNNIASWFT	RRPQGLPNNTASWFT	0.533253	0.584355
YNLKWN	YNL-WN	0.520244	0.765309
AGCLIGAEHVDTSYECDI	AGCLIGAEHVNNSYECDI	0.503905	0.56813
GFMKQYGECLGDINARDL	GFIKQYGDCLGDIAARDL	0.471939	0.506817
ANKEGIVWVATEGAL	ANKDGIIWVATEGAL	0.367723	0.404796
WNPDDY	WNADLY	0.355018	0.584726
PDDYGG	PDDFTG	0.334887	0.527287
TWLTYHGAIKLDDKDPQF	TWLTYTGAIKLDDKDPNF	0.27017	0.529675
DEVNQI	DEVRQI	0.18504	0.187797
SSKRFQPFQQFGRDV	SNKKFLPFQQFGRDI	0.098384	0.119472
NHDSPDAEL	NHTSPDVDL	0.067808	0.17889
TKQYNVTQAF	TKAYNVTQAF	0.054818	0.171488
VKQMYKTPTLKYFGGFNF	VKQIYKTPPIKDFGGFNF	0.018685	0.135681
QKRTATKQYNVTQAF	QKRTATKAYNVTQAF	0.004891	0.037776

It is worth noting that in general predicting immunogenicity of given a peptide is challenging and not a fully solved problem, and therefore current models for predicting immunogenicity are suboptimal. iPred is also not an exception. In fact, we could see that a substantial number of IEDB immunogenic peptides were scored < 0.5 (the threshold score used to classify immunogenic vs non-immunogenic). This led us to ask whether we can gather any other evidence of either immunogenicity or cross-reactivity.

### *De novo* search of immunogenic peptides in 2019-nCoV proteome

As a complementary reciprocal approach, we conducted a *de novo* search of immunogenic peptides against the 2019-nCov proteome sequence. We scanned 9-mers from 2019-nCoV proteome with a window of 9 amino acids and step length of 1 amino acid (9613 in total). The immunogenicity of 9-mer peptides were predicted using iPred and MHC presentation scores were gauged using NetMHCpan 4.0 ^6^ for various HLA types. In this task, we focused on haplotypes common in Chinese and European populations, which include HLA-A*02:01, HLA-A*01:01, HLA-B*07:02, HLA-B*40:01 and HLA-C*07:02 alleles (Data Table 3 ^4^).

**Figure 3.**
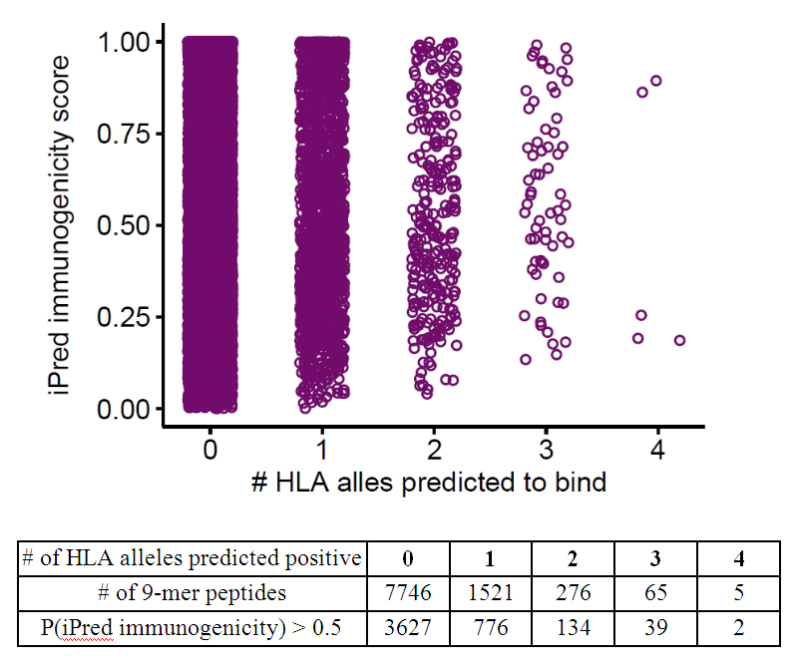
*De novo* search of 9-mer 2019-nCoV peptides with MHC presentation and immunogenicity potential. The MHC binding was predicted for HLA-A*02:01, HLA-A*01:01, HLA-B*07:02, HLA-B*40:01 and HLA-C*07:02 alleles by NetMHCpan 4.0 and immunogenicity was predicted by iPred.

Based on MHC presentation and immunogenicity prediction, we detected 5 peptides predicted to bind 4 different HLA alleles of which 2 had strong immunogenicity scores ( Figure 3). For those 65 strong binders to 3 different HLA types, 39 had immunogenicity scores ≥ 0.5 ( Table 3). Collectively this analysis suggests a number of 9-mer immunogenic candidates for further experimental validation.

**Table 3.** 2019-nCoV 9-mer peptides predicted to bind 4 different HLA alleles by NetMHCpan 4.0, and those predicted to bind ≥ 3 HLA alleles and immunogenicity score ≥ 0.9 by iPred. For different alleles, 0 denotes non-binding and 1 denotes binding predicted for specific HLA allele.

Antigen.epitope	Imm.prob	A0101.NB	A0201.NB	B0702.NB	B4001.NB	C0702.NB	Total binding HLA
VQMAPISAM	0.893938	0	1	1	1	1	4
AMYTPHTVL	0.862427	0	1	1	1	1	4
TLDSKTQSL	0.254998	1	1	1	0	1	4
KVDGVVQQL	0.191786	1	1	1	0	1	4
KVDGVDVEL	0.18632	1	1	1	0	1	4
MADQAMTQM	0.991227	1	0	1	0	1	3
LEAPFLYLY	0.983072	1	0	0	1	1	3
RTAPHGHVM	0.972153	1	0	1	0	1	3
IPFAMQMAY	0.961569	1	0	1	0	1	3
FLTENLLLY	0.951715	1	1	0	0	1	3
YLQPRTFLL	0.947743	1	1	0	0	1	3
MMISAGFSL	0.941318	0	1	1	0	1	3
ATLPKGIMM	0.926603	1	0	1	0	1	3

### Immunogenicity of 2019-nCoV peptides to 1G4 CD8+ TCR molecule

While our *de novo* candidates are appealing shortlisted targets for experimental validation, it does not provide information about target T cell receptors (TCRs). We therefore set out to interrogate the possibility of cross reactivity with one well-studied TCR.

T cell cross-reactivity has been instrumental for the T cell immunity against both tumor antigens and external pathogens. In that regard, a number of T cells have been extensively characterized including 1G4 CD8+ TCR, which is known to recognize the ‘SLLMWITQC’ peptide presented by HLA-A*02:01. We therefore set out to leverage the data from a recently published study ^7^ and exploit the possibility of cross reactivity of this TCR to any 2019-nCoV peptide.

**Figure 4.**
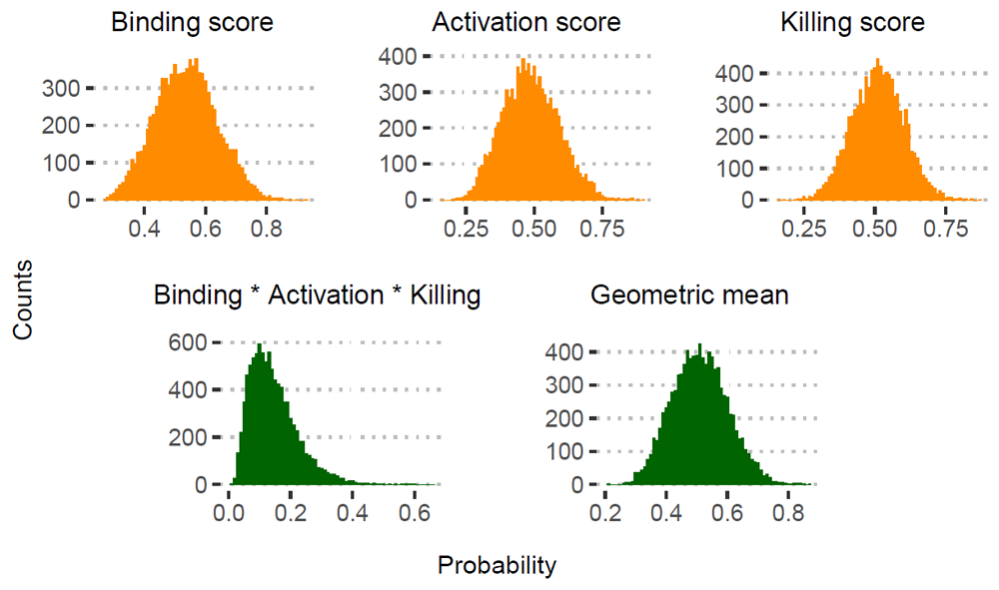
Distribution of 1G4 TCR positional weight matrix scores for 2019-nCoV peptides. The positional weight matrices were obtained from 7 and 9613 9-mers generated from 10 2019-nCoV ORFs were computed for their TCR recognition potential.

Here, we scanned all 9-mers from the 2019-nCoV proteome (9613 peptides) with Binding, Activating and Killing Position Weight Matrices (PWM, see the method section) and associated each peptide with the geometric mean of these three assays as a measure of immunogenicity (Data Table 4 ^4^). The distributions of binding, activation and killing scores along with their multiplicative score and geometric mean are illustrated in Figure 4. Based on geometric mean, we observed 20 2019-nCoV peptides with a score > 0.8 and 516 peptides > 0.7. The 9-mer peptides with geometric mean > 0.7 and positive HLA-A*02:01 binding prediction by NetMHCpan 4.0 are listed in Table 4.

**Table 4.** 2019-nCoV 9-mer peptides with geometric mean ≥ 0.7 by 1G4 TCR positional weight matrix and predicted positive to bind HLA-A*02:01 by NetMHCpan 4.0 (Rank = NetMHCpan rank).

Peptide	Binding score	Activation score	Killing score	geoMean	Rank	Binder
RIMTWLDMV	0.866377428	0.853995	0.776303	0.831249	0.3481	SB
ALNTLVKQL	0.802453741	0.75073	0.785957	0.779413	0.6159	WB
LLLDRLNQL	0.809895414	0.7752	0.741096	0.774888	0.0423	SB
MIAQYTSAL	0.766262499	0.789511	0.749477	0.768242	0.9238	WB
VLSTFISAA	0.799672451	0.756117	0.687278	0.746239	0.536	WB
NVLAWLYAA	0.761297552	0.686117	0.739944	0.728423	1.4457	WB
RLANECAQV	0.783161706	0.719705	0.680504	0.726572	0.2049	SB
KLLKSIAAT	0.748896679	0.708996	0.697463	0.718118	1.0923	WB
QLSLPVLQV	0.70128376	0.715259	0.708405	0.708293	0.4768	SB
VQMAPISAM	0.729320768	0.698514	0.689612	0.705612	1.4677	WB
LLLTILTSL	0.7131709	0.715194	0.680064	0.702623	0.2712	SB
SVLLFLAFV	0.736972762	0.690855	0.679534	0.70202	1.1449	WB
LMWLIINLV	0.727847374	0.681119	0.694007	0.700717	1.304	WB

We further analysed the MHC binding propensities and gathered peptides not only predicted positive by NetMHCpan but also to have leucine (L) and valine (V) in anchor positions 2 (P2) and 9 (P9) respectively. Previous studies have shown that for MHC-I HLA-A02:01 specific peptides, 9-mer peptides with leucine at P2 and valine at P9 are preferably presented on the surface of HLA-A02:01 ^8^. Looking at the LV peptide, we identified 44 2019-nCoV peptides of which 2 peptides had immunogenicity score > 0.7 and 12 peptides > 0.6 ( Table 5). Thus, here we provide the list of peptides that are potential targets for 1G4 TCR recognition for subjects with HLA-A02:01 haplotype.

**Table 5.** 2019-nCoV 9-mer peptides having leucine-valine in anchor positions. Peptides have geometric mean ≥ 0.6 and ≤ 0.7 (for those ≥ 0.7, refer to Table 4) by 1G4 TCR positional weight matrix and predicted positive for HLA-A*02:01 binding by NetMHCpan 4.0 (Rank = NetMHCpan rank).

Peptide	Binding score	Activation score	Killing score	geoMean	Rank	Binder
TLMNVLTLV	0.723687	0.658986	0.652178	0.677534	0.0444	SB
QLEMELTPV	0.711291	0.651003	0.608605	0.655625	1.6769	WB
MLAKALRKV	0.668756	0.610664	0.65968	0.645854	0.3524	SB
GLFKDCSKV	0.675952	0.632375	0.594753	0.633494	0.2677	SB
ALSKGVHFV	0.652549	0.604952	0.586236	0.613954	0.0425	SB
YLNTLTLAV	0.624147	0.610826	0.575445	0.603119	0.0453	SB

## Discussion

In this study we provide a profile of computationally predicted immunogenic peptides from 2019-nCoV for functional validation and potential vaccine developments. We are fully aware that an effective vaccine development will require a very thorough investigation of immune correlates to 2019-nCoV. However, due to the emergency and severity of the outbreak as well as the lack of access to samples from infected subjects, such approaches would not serve the urgency. Therefore, computational prediction is instrumental for guiding biologists towards a quick and cost-effective solution to prevent the spread and ultimately help eliminate the infection from the individuals.

With a rising global concern of novel coronavirus outbreak, numerous research groups have started to investigate and publish their findings. At the time of preparing this manuscript, we became aware of a similar study conducted in comparing 2019-nCoV proteome with SARS CoV immunogenic peptides ^9^. Our *in silico* approach takes the search beyond presenting only common immunogenic peptide between SARS and 2019-nCoV and provides the experimental community with a more comprehensive list including *de novo* and cross reactive candidates. On the other hand, considering the fact that two studies have been accomplished independently with distinct approaches, this serves to demonstrate a high level of confidence in reproducing the results. Reproducibility of computational prediction is always of high importance and becomes even more significant under urgent scenarios as of this outbreak.

Our study also suggests the need for further efforts to develop accurate predictive models and algorithms for the characterization of immunogenic peptides.

In this study, we provide potential immunogenic peptides from 2019-nCoV for vaccine targets that i) have been characterized immunogenic by previous studies on SARS CoV, ii) have high degree of similarity with immunogenic SARS CoV peptides and iii) are predicted immunogenic by combination of NetMHCpan and iPred/1G4 TCR positional weight matrices. Given the limited time and resources, our work serves as a guide to save time and cost for further experimental validation.

## Method

### Data acquisition

2019-nCoV open reading frame sequences were downloaded from NCBI ( MN908947.3). All sequences subjected for analysis are deposited in GitHub repository.

### Data analysis

All subsequent analyses have been conducted in R 3.6.1.

### Sequence similarity comparison

The sequence similarity between 2019-nCoV open reading frames and previously characterized immunogenic peptides in IEDB was analysed by local alignment using R ‘pairwiseAlignment’ function from Biostrings v2.40.2 package. The local alignment utilized BLOSUM62 matrix, gapOpening of 5 and gapExtension of 5. The alignment score was normalized by length of target peptides.

In extracting peptides with the exact sequence alignment with epitope sequences in IEDB, only peptides with more than 3 amino acids in length were shortlisted.

### Immunogenicity prediction

We have used iPred ^5^ to predict immunogenicity of each given peptide. Briefly, iPred employs peptides’ length and physicochemical properties of amino acids modelled by sums of ten Kidera factors and associates a score to each peptide reflecting its likelihood of recognition by a T cell.

### Predicting presentation by MHCs

In order to predict peptide binding to MHC we used NetMHCpan V4 ^6^. This version of NetMHCpan that comes with a number of improvements, incorporate both eluted ligand and peptide binding affinity data into a neural network model to predict MHC presentation of each given peptide.

### Predicting cross reactivity to 1G4 TCR

To gauge the level of 1G4 TCR cross-reactivity to list of 2019-nCoV virus, we have leveraged the data from a recently published study ^7^. 1G4 or NY-ESO-1-specific TCR is a very well-studied and clinically efficacious TCR which recognize the peptide ‘SLLMWITQC’ presented by HLA-A*02:01. Karapetyan *et al.* have recently provided data from three experimental assays reflecting Binding, Activating and Killing upon each mutation at each position of all possible 9-mers using these three datasets. In a similar way to the original paper, we trained three Position Weight Matrices named B, A and K respectively from Binding, Activating and Killing assay. We defined the cross-reactivity score of a given 9-mer sequence as the geometric mean of B, A and K.

We then scanned 2019-nCoV virus protein sequence with each of B, A and K PWMs and associated each of 9613 9-mers with a cross reactivity score. At the same we utilized NetMHCpan and associated each 9-mer with its presentation score. Our final list of cross-reactive candidate peptides were those with a cross-reactivity sore >= 0.8 and reported as strong binders from NetMHCpan and have ‘L’ and ‘V’ amino acids at anchor positions. The custom R codes are accessible from GitHub repository (see software availability ^4^).

## Software availability

Replication code: https://github.com/ChloeHJ/Vaccine-target-for-2019-nCoV.git

Archived source code at time of publication: http://doi.org/10.5281/zenodo.3676908 ^4^

License: Creative Commons Attribution 4.0 International

## Data availability

### Source data

2019-nCoV open reading frame sequences were downloaded from NCBI ( MN908947.3).

### Underlying data

Zenodo: *In silico* identification of vaccine targets for 2019-nCoV (Data tables). http://doi.org/10.5281/zenodo.3676886 ^10^

This project contains the following underlying data:

– Table1 nCoV peptides having exact match with immunogenic SARS CoV peptides.xlsx (Table of nCoV peptides having exact match with immunogenic SARS CoV peptides)
– Table2 nCoV peptides with high sequence similarity with immunogenic IEDB peptides.csv (Table of peptides with high sequence similarity with immunogenic IEDB peptides)
– Table3 *de novo* search on 9-mer nCoV for immunogenic peptides by NetMHCpan and iPred.csv (Table of results of *de novo* search on 9-mer nCoV for immunogenic peptides by NetMHCpan and iPred)
– Table4 *de novo* search on 9-mer nCoV for immunogenic peptides by NetMHCpan and PWM.xlsx (Table of results of *de novo* search on 9-mer nCoV for immunogenic peptides by NetMHCpan and PWM)

Data are available under the terms of the Creative Commons Attribution 4.0 International license (CC-BY 4.0).
