# Supplementary material for: In silico identification of vaccine targets for 2019-nCoV
Source: F1000Res. 2020 Apr 14;9:145. Originally published 2020 Feb 25. [Version 2] doi: 10.12688/f1000research.22507.2 (PMC7111504; doi:10.12688/f1000research.22507.2)
Supplement: Peer review history

# Peer review: Reviewer response for version 2

**DOI:** 10.5256/f1000research.25726.r61739

**Published:** 2020-04-14

**Authors:** Angelika Riemer, Maria Bonsack

**Affiliations:** 1 Immunotherapy and Immunoprevention, German Cancer Research Center (DKFZ), Heidelberg, Germany; 2 Molecular Vaccine Design, German Center for Infection Research (DZIF), partner site Heidelberg, Germany

The authors present a list of potential 2019-nCoV vaccine candidates based on an *in silico* prediction approach. Overall, they present 28 2019-nCoV-derived peptides that exactly match epitopes of SARS-CoV, and additional 48 2019-nCoV peptides that overlap with already known immunogenic epitopes reported in IEDB. The latter have been evaluated using immunogenicity prediction resulting in 22 2019-nCoV peptides with higher immunogenicity scores than their IEDB counterpart. Additional analysis of encoded 2019-nCoV peptides using HLA binding likelihood and immunogenicity scores predicted by NetMHCpan 4.0 and iPred, respectively, showed 13 additional vaccine candidates regarding prevalent European and Asian HLA-types. Finally, the authors modeled possible cross-reactivity of a well described HLA-A*02:01-NY-ESO-1-peptide-reactive TCR to 2019-nCoV peptides with predicted HLA binding affinity and found 19 hits. With this, authors provide a shortlist of potential candidates for development of a 2019-nCoV vaccine applicable to a broad population.

 The paper would benefit from clearer labelling of the figures and tables, and more extensive description of shown results in the figure legends. Column titles should be consistent among tables and figures, and ideally not consist of the internally used abbreviations but be self-explanatory.

 In Figure 1A, the axis labeling (Counts and Alignment Score) seems to be switched. 1B: Adding a note that these are non-exact peptides with alignment score >=4 would be helpful. In Figure 2, 45 peptides are shown, but 48 peptides are mentioned in the text. Please clarify.

 General remarks: It would be better to use “encoded proteins” than the term “proteome”, as the data derives from 10 ORF sequences. The authors should indicate in the methods the employed thresholds of the used predictors to label a peptide a binder or immunogenic. These thresholds could also be shown in the figures where appropriate. As iPred only uses MHC binding likelihood and amino acid qualities to predict peptide presentation which is not very different from general NetMHCpan approach (which is also implemented in this tool), the authors should discuss why they used iPred instead of well described tools such as NetTepi or NetCTL and NetCTLpan. It does also not come across why an anti-NY-ESO-1 TCR should be cross-reactive to any 2019-nCoV peptide?

Is the work clearly and accurately presented and does it cite the current literature?

Yes

If applicable, is the statistical analysis and its interpretation appropriate?

Not applicable

Are all the source data underlying the results available to ensure full reproducibility?

Yes

Is the study design appropriate and is the work technically sound?

Yes

Are the conclusions drawn adequately supported by the results?

Yes

Are sufficient details of methods and analysis provided to allow replication by others?

Partly

Reviewer Expertise:

Therapeutic cancer vaccination; T cell epitope prediction,  identification and validation; Immunopeptidomics

We confirm that we have read this submission and believe that we have an appropriate level of expertise to confirm that it is of an acceptable scientific standard.

# Peer review: Reviewer response for version 1

**DOI:** 10.5256/f1000research.24839.r60502

**Published:** 2020-03-31

**Authors:** Raffaele De Palma

**Affiliations:** 1 DIMI, Department of Internal Medicine, University of Genova, Genova, Italy; 2 IBBC (Istituto di Biochimica e Biologia Cellulare), CNR-Napoli, Naples, Italy

The Authors use an *in silico* approach to identify antigenic peptides derived from 2019-nCoV. First, they screened ten open reading frame of 2019-nCoV sequence used the IEDB database, finding a series of peptides potentially immunogenic. The first piece of data relies on the identification of 28 peptides that were exactly matching SARS CoV peptides. Moreover, using combinatory approaches, modelling HLA and TCR binding, they identified 13 peptides potentially able to bind a given TCR in HLA A2 restriction fashion, and cutting a list of peptides able to bind several HLA alleles and characterizing several peptides that may be de novo candidates or crossreactive peptides to be used either to study immune response to 2019-nCoV or to set a vaccine.

Is the work clearly and accurately presented and does it cite the current literature?

Yes

If applicable, is the statistical analysis and its interpretation appropriate?

Yes

Are all the source data underlying the results available to ensure full reproducibility?

Yes

Is the study design appropriate and is the work technically sound?

Yes

Are the conclusions drawn adequately supported by the results?

Yes

Are sufficient details of methods and analysis provided to allow replication by others?

Yes

Reviewer Expertise:

T cell responses in Health and Diseases.  Modulation of Immune Responses. Clinical Immunology.  Vaccines

I confirm that I have read this submission and believe that I have an appropriate level of expertise to confirm that it is of an acceptable scientific standard.

# Peer review: Reviewer response for version 1

**DOI:** 10.5256/f1000research.24839.r60504

**Published:** 2020-03-05

**Authors:** Katalin Wilkinson

**Affiliations:** 1 Tuberculosis Laboratory, The Francis Crick Institute, London, UK; 2 Institute of Infectious Disease and Molecular Medicine, University of Cape Town, South Africa

This is an important manuscript identifying potential vaccine targets for 2019-nCoV, using computational prediction. In the absence of patient samples, such approaches are instrumental to guide towards quick and efficient identification of vaccine candidates. The authors used 10 open reading frame sequences of 2019-nCoV deposited at NCBI and conducted sequence alignment against immunogenic peptides deposited in the Immune Epitope Database and Analysis Resource (IEDB) database. They identified 28 peptides with sequences matching exactly to severe acute respiratory syndrome-related coronavirus (SARS CoV), that have previously been characterised as immunogenic by T cell assays. These findings are very promising and have the added benefit of potentially developing a vaccine against both SARS and COVID-19.

 Additional peptides were identified to most likely bind common Chinese and European HLA alleles and have high immunogenicity potential. The authors provide a shortlist of peptides as potential vaccine candidates. While this manuscript presents a good model for identifying such targets, a comment should be included in the discussion about the necessity of expanding the analysis to include wider HLA allele types, considering that the virus is likely to spread worldwide.

 Minor comment: Please explain the methods and analysis in greater details, including the following terms:

1. ‘Towards target peptide length > 3’.
2. ‘Normalized alignment scores’ (and their scale, such as the significance of 4 for the data presented in Figure 1).
3. The importance of leucine (L) and valine (V) in anchor positions for MHC binding (for data presented in Table 5).

Is the work clearly and accurately presented and does it cite the current literature?

Yes

If applicable, is the statistical analysis and its interpretation appropriate?

Not applicable

Are all the source data underlying the results available to ensure full reproducibility?

Yes

Is the study design appropriate and is the work technically sound?

Yes

Are the conclusions drawn adequately supported by the results?

Yes

Are sufficient details of methods and analysis provided to allow replication by others?

Partly

Reviewer Expertise:

T cell immunology

I confirm that I have read this submission and believe that I have an appropriate level of expertise to confirm that it is of an acceptable scientific standard.

# Response

**Published:** 2020-04-07

**Authors:** Hashem Koohy

**Affiliations:** Oxford University

Dear Prof Wilkinson,

 Thank you so much for your time and valuable suggestions. In our new and revised version we have taken your suggestions into consideration.

 Best regards

 Hashem Koohy
